# Depression and risk of hospital readmission in older patients with heart failure: a systematic review and meta-analysis

**DOI:** 10.3389/fcvm.2026.1917904

**Published:** 2026-09-18

**Authors:** Sihan Wu, Heping Yuan, Hexin Zhu, Yating Zhang, Xiumei Qi

**Affiliations:** 1 School of Nursing, Shandong First Medical University, Jinan, China; 2 School of Nursing, Shandong University of Traditional Chinese Medicine, Jinan, China; 3 School of Nursing, Shandong Medical and Pharmaceutical University, Yantai, China; 4 Department of Education, The First Affiliated Hospital of Shandong First Medical University (Shandong Provincial Qianfoshan Hospital), Jinan, China

**Keywords:** depression, heart failure, meta-analysis, older adults, readmission, systematic review

## Abstract

**Background:**

Depression is common among patients with heart failure (HF) and may adversely affect clinical outcomes. However, its association with hospital readmission in older patients with HF remains incompletely understood.

**Methods:**

A systematic review and meta-analysis was conducted in accordance with PRISMA guidelines. PubMed, Embase, Web of Science, CNKI, and Wanfang databases were searched from inception to 31 December 2025. Cohort studies examining the association between depression and readmission outcomes in patients with HF were included. Pooled hazard ratios (HRs) and 95% confidence intervals (CIs) were calculated using a random-effects model.

**Results:**

A total of 29 studies were included, of which 13 were eligible for meta-analysis. Depression was associated with a significantly increased risk of readmission and adverse outcomes (HR = 1.76, 95% CI: 1.30–2.40). In studies using continuous measures, higher depressive symptom scores were also associated with increased risk (HR = 1.04, 95% CI: 1.02–1.07). Subgroup analyses suggested a stronger association in studies with longer follow-up, although between-group differences were not statistically significant. Sensitivity analyses confirmed the robustness of the findings.

**Conclusions:**

Depression is associated with an increased risk of readmission in patients with heart failure. Routine assessment of depressive symptoms may improve risk stratification and support targeted post-discharge management, particularly in older populations.

## Introduction

Heart failure (HF) is a complex clinical syndrome resulting from structural or functional cardiac abnormalities and remains a leading cause of morbidity, mortality, and reduced quality of life worldwide. Despite advances in therapeutic strategies, HF continues to impose a substantial burden on healthcare systems due to its high rates of hospitalization and healthcare resource utilization (1). Recurrent hospitalizations are a major challenge in HF management and are associated with increased morbidity, mortality, and healthcare utilization. Contemporary data suggest that nearly one in four patients hospitalized for HF are readmitted within 30 days after discharge, and the risk of rehospitalization remains high during the first several months after discharge (2). Older patients with HF are particularly vulnerable to poor outcomes. Evidence from a nationwide registry study in China including 230,637 discharged patients from 572 hospitals showed that the all-cause mortality rates at 30 days and 1 year after discharge were 2.4% and 13.7%, respectively. The study further demonstrated that both mortality and readmission risks increased significantly with age, highlighting the importance of identifying modifiable risk factors in elderly patients with HF (3).

Depression is one of the most common psychological comorbidities among patients with heart failure (HF) (4). Previous studies have shown that clinically significant depressive symptoms are present in approximately 20%–40% of patients with HF, and the prevalence may be even higher in those with more advanced disease or functional impairment (5). Patients with HF may be particularly vulnerable to depression because of persistent symptoms, impaired physical functioning, repeated hospitalizations, and reduced quality of life (4). In clinical practice, depression may negatively affect multiple aspects of HF care. Depressive symptoms have been associated with poorer self-care behaviors, reduced treatment adherence, lower motivation for symptom monitoring, and decreased participation in disease management (6). In addition to its impact on self-management, depression has also been linked to adverse clinical outcomes in patients with HF. Previous systematic reviews and meta-analyses have consistently shown that depression is associated with increased mortality, hospitalization, and other adverse outcomes in patients with heart failure (7–9). Recent evidence has further highlighted the close relationship between cardiovascular comorbidities and psychological distress in older adults, emphasizing that depression frequently coexists with cardiovascular disease and may adversely affect clinical outcomes (10). Taken together, these findings suggest that depression may represent an important and potentially modifiable prognostic factor in HF management.

A growing number of studies have examined the relationship between depression and post-discharge outcomes in patients with heart failure (5). Existing cohort studies have generally suggested that depression may be associated with an increased risk of hospitalization, readmission, and other adverse clinical outcomes in this population (11). Previous meta-analyses by Rutledge et al. (7) and Sokoreli et al. (8) reported an association between depression and adverse outcomes in patients with heart failure. However, these reviews were based primarily on studies published more than a decade ago and did not specifically focus on hospital readmission in older populations or synthesize evidence from studies reporting continuous depression symptom scores. Since then, several large cohort studies have provided additional evidence on the association between depression and hospital readmission in patients with heart failure. Nevertheless, variations in study design, depression assessment tools, follow-up duration, and outcome definitions may limit the comparability of individual findings. Therefore, we conducted an updated systematic review and meta-analysis to evaluate the association between depression and hospital readmission among older patients with heart failure. This review incorporates recently published evidence and synthesizes data from both categorical and continuous measures of depression.

## Methods

### Search strategy

This systematic review and meta-analysis was conducted in accordance with the Preferred Reporting Items for Systematic Reviews and Meta-Analyses (PRISMA) 2020 statement. A literature search was performed in PubMed, Embase, Web of Science, China National Knowledge Infrastructure (CNKI), and Wanfang databases from their inception to 31 December 2025 to identify studies examining the association between depression and the risk of hospital readmission among older patients with heart failure. The detailed search strategies for all databases are presented in Supplementary Table S1. In addition, the reference lists of the included studies and relevant reviews were manually screened to identify additional eligible studies. Preprint servers were not searched.

### Eligibility criteria

Studies were included in this review if they enrolled older adults with heart failure, as defined by the original study authors, evaluated depression or depressive symptoms as the exposure, and reported hospital readmission outcomes, including heart failure–related readmission, all-cause readmission, or composite outcomes involving readmission. Both prospective and retrospective cohort studies were considered eligible.

Studies were excluded if they were reviews, editorials, case reports, conference abstracts, or lacked sufficient data to assess the association between depression and readmission outcomes. When multiple publications originated from the same or overlapping cohorts, all eligible publications were retained in the qualitative synthesis when they reported different outcomes, follow-up periods, or analyses. However, only one independent effect estimate from each cohort was included in each pooled analysis to avoid double-counting participants. When multiple reports from the same cohort were available, the study providing the most complete multivariable-adjusted data was retained for quantitative synthesis. No study contributed more than one effect estimate to the same pooled analysis.

For quantitative synthesis, studies were included only when multivariable-adjusted hazard ratios (HRs) with corresponding 95% confidence intervals (CIs) were available or could be calculated. Unadjusted estimates were not pooled in order to minimize the potential influence of confounding.

### Study selection

Study selection was conducted in accordance with the predefined eligibility criteria. Two reviewers independently screened titles and abstracts of all retrieved records to identify potentially eligible studies. Full texts of potentially relevant articles were then reviewed for eligibility.

Disagreements between reviewers were resolved through discussion, and when necessary, consultation with a third reviewer was undertaken to reach consensus.

The study selection process was documented using a PRISMA flow diagram (Figure 1) (12).

**Figure 1:**
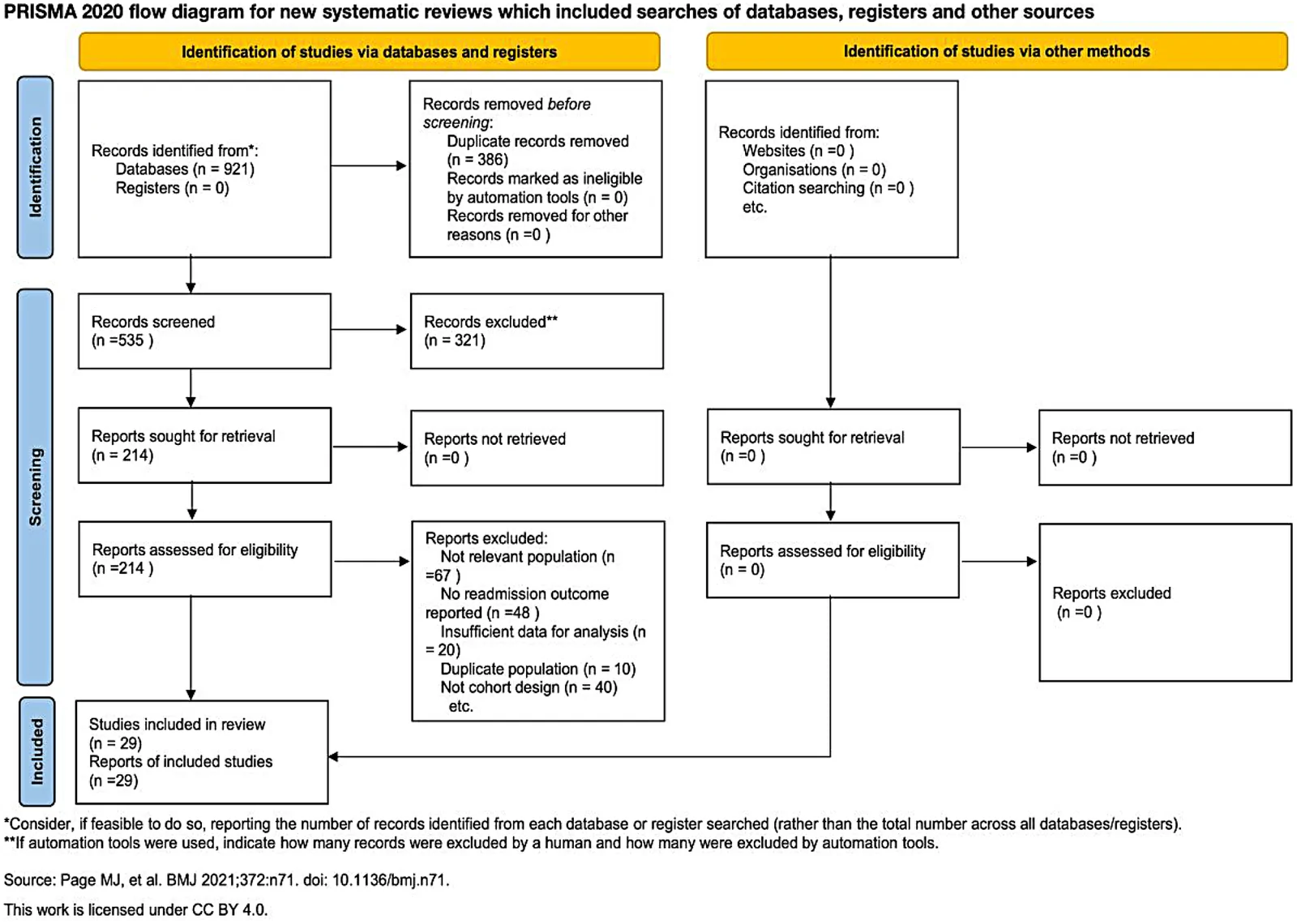
PRISMA 2020 flow diagram of study selection.

### Data extraction

Data were extracted independently by two reviewers using a predefined form. Information collected from each study included author, publication year, country, study design, sample size, participant characteristics, methods used to assess depression, follow-up duration, outcome definitions, reported effect estimates with their 95% confidence intervals and the covariates included in multivariable adjustment models, when available.

When multiple estimates were reported, adjusted hazard ratios derived from multivariable models were extracted whenever available. Depression assessment methods were also classified as self-reported depression scales or clinical/administrative diagnosis for subgroup analyses. Any discrepancies between reviewers were resolved through discussion until agreement was reached.

### Quality assessment

The methodological quality of the included studies was assessed using the Newcastle–Ottawa Scale (NOS) (13).

This tool evaluates the quality of cohort studies across three domains: selection of study groups, comparability of cohorts, and ascertainment of outcomes. The total NOS score ranges from 0 to 9 points, with higher scores indicating better methodological quality. In this study, a score of 7 or above was considered indicative of high methodological quality.

### Statistical analysis

Adjusted hazard ratios (HRs) and corresponding 95% CIs were pooled using a random-effects model. Statistical heterogeneity was assessed using Cochran's *Q* test and the *I*^2^ statistic, with *I*^2^ values above 50% indicating substantial heterogeneity.

Subgroup analyses were conducted based on follow-up duration, outcome type, and depression assessment method (self-reported depression scales vs. clinical/administrative diagnosis) to explore potential sources of heterogeneity.

Sensitivity analyses were performed using a leave-one-out method to evaluate the robustness of the pooled estimates. In addition, a sensitivity analysis restricted to studies with a mean age ≥65 years was conducted to assess the robustness of the primary findings.

Publication bias was assessed visually using funnel plots.

All analyses were conducted using Review Manager (RevMan) version 5.4.1.

## Results

### Study selection

A total of 921 records were identified through database searching. After removal of 386 duplicate records, 535 records remained for title and abstract screening. Of these, 321 records were excluded.

A total of 214 full-text articles were assessed for eligibility. After full-text review, 185 reports were excluded for the following reasons: not relevant population (*n* = 67), no readmission outcome reported (*n* = 48), insufficient data for analysis (*n* = 20), duplicate population (*n* = 10), and non-cohort design (*n* = 40).

Finally, 29 studies were included in the qualitative synthesis, of which 13 were included in the quantitative meta-analysis.

### Study characteristics

A total of 29 studies were included in the qualitative synthesis, of which 13 were included in the quantitative meta-analysis. All studies employed cohort designs, including both prospective and retrospective cohorts.

The sample sizes varied considerably across studies. Participants were patients with heart failure, and several studies specifically focused on older populations. The duration of follow-up ranged from several months to several years.

Depression was assessed using a range of validated instruments. The most commonly used tools included the Patient Health Questionnaire (PHQ-9) (14), the Hospital Anxiety and Depression Scale (HADS) (15), and the Beck Depression Inventory (BDI) (16). In some studies, depression was identified through clinical diagnosis or structured assessments.

With regard to outcomes, most studies reported all-cause readmission or heart failure–related readmission, while a small number used composite outcomes, such as readmission combined with mortality. In addition, most studies adjusted for potential confounding factors in multivariable analyses, including age, sex, comorbidities, and indicators of disease severity.

Further details of the included studies are summarized in Table 1.

**Table 1:** Characteristics of the included studies.

Study	Country	Study design	Sample size	Mean age	Depression assessment	Outcome	Effect estimate
Jiang et al. (17)	USA	Prospective cohort study	357	Mean age 64 ± 13 years	BDI + modified DIS	All-cause mortality and readmission at 3 months and 1 year	Adjusted OR
Freedland et al. (18)	USA	Prospective cohort study	400	Mean age 58.4 ± 13.1 years	PHQ-9	Multiple all-cause readmissions over 2 year	Adjusted HR
Lesman-Leegte et al. (19)	Netherlands	Prospective cohort study	958	Mean age 71 ± 11 years; adults hospitalized for HF	CES-D	HF readmission and all-cause mortality over 18 months	Adjusted HR
Lu et al. (20)	USA	Prospective cohort study	611	Mean age 66 ± 15 years	Documented diagnosis of depression	30-day and overall HF readmission	Adjusted HR
Ma et al. (21)	China	Longitudinal observational study	432	Mean age 68 ± 8.6 years	CES-D (>10)	6-month rehospitalisation	Adjusted OR
Patel et al. (11)	USA	Retrospective cohort study	3,500,570	Mean age 72.8 ± 14.8 years	Administrative diagnosis/database-defined depression	30-day and 90-day all-cause readmission	Adjusted HR
Piepenburg et al. (22)	Germany	Prospective cohort study	852	>18 years; mean age 67.6 ± 12.1 years	PHQ-2/PHQ-9	18-month all-cause rehospitalization and mortality	HR
Piepenburg et al. (23)	Germany	Prospective cohort study	852	Mean age 67 ± 13 years	PHQ-9	540-day all-cause mortality and rehospitalization	HR
Reeves et al. (24)	USA	Longitudinal cohort study	1,260	Mean age 63.57 ± 13 years	PHQ-9 (cutoff = 10)	12-month all-cause mortality and cardiac rehospitalization	Adjusted HR
Setoguchi et al. (25)	Japan	Retrospective cohort study	310	Mean age 78 ± 11 years (HFpEF/HFrEF combined)	CES-D (Japanese version), medical record–based definition	1-year HF readmission	Adjusted HR
Song et al. (26)	South Korea	Prospective cohort study	165	Mean age 62.2 ± 12.5 years	BDI	6-month HF rehospitalization	Adjusted HR
Suzuki et al. (27)	Japan	Prospective cohort study	139	Mean age 67.6 ± 12.9 years	BDI	1-year HF-related readmission	Adjusted HR
Mao et al. (28)	China	Retrospective cohort study	160	Mean 75 ± 9 years	PHQ-9	1-year cardiovascular-related readmission	Adjusted HR
Moraska et al. (29)	USA	Prospective cohort study	402	Mean 73 ± 13 years	PHQ-9	Hospitalizations, ED visits, outpatient visits, all-cause mortality	Adjusted HR
Suzuki et al. (30)	Japan	Prospective observational study	976	Mean 66 ± 13 years	PHQ-2	Composite of all-cause death or hospitalization due to worsening HF	Adjusted HR
Albert N et al. (31)	USA	Registry-based observational study	48,612	Mean 73.2 years	Medical record-documented history of depression	Length of stay, mortality, emergency care, rehospitalization	Adjusted OR/HR
Alhurani et al. (32)	USA	Longitudinal cohort study	1,260	Mean 63.57 ± 13 years	PHQ-9 (cutoff ≥ 10)	All-cause mortality and cardiac rehospitalization	Adjusted HR
Beach et al. (33)	USA	Secondary analysis of a randomized trial/prospective cohort	172	Mean 62.1 ± 12.4 years	PHQ-9	6-month cardiac-related readmission; composite of cardiac readmission or all-cause mortality	Adjusted HR
Cha et al. (34)	USA	Secondary analysis	1,002	Mean 64.3 ± 12.7 years	PHQ-9	Death or rehospitalization	Adjusted HR
Chavanon et al. (35)	USA/Germany	Observational cohort study	394	Mean 65.9 ± 13.2 years	PHQ-2 screening and PHQ-9	All-cause rehospitalization	Adjusted HR
Freedland et al. (36)	USA	Prospective cohort study	662	Mean 66.2 ± 11.9 years; age ≥40 years	DSM-IV depression diagnosis and depression symptom count (Diagnostic Interview Schedule)	Multiple all-cause rehospitalizations over 1 year	Adjusted HR
Oud et al. (37)	Netherlands	Cross-sectional cohort study	157	Median age 79 years; age ≥65 years	GDS-2 and GDS-15 (>5)	Overall readmission, HF-related readmission, and mortality within 6 months	Not reported
Redwine et al. (38)	USA	Prospective cohort study	18	Mean age 62 years	HAM-D	Cardiac-related hospitalization and/or death over 2 years	OR(continuous depression score)
Xu et al. (39)	USA	Mixed-methods study	127	Mean age 58.1 ± 13.6 years	CES-D-10 (≥10)	30-day rehospitalization and delayed decision making	Adjusted OR
Zhang et al. (40)	China	Prospective cohort study	381 (training); 170 (external validation)	Aged ≥60 years; mean age 72.74 years	HADS (depression subscale)	30-day all-cause readmission	Adjusted OR
Braunstein et al. (41)	USA	Cross-sectional study	122,630	Age ≥65 years; mean age 79.0 years	Depression/affective disorder identified from Medicare claims/ICD codes	Preventable hospitalization and mortality within 1 year	Adjusted OR/HR
Jaarsma et al. (42)	Netherlands	Secondary analysis of a randomized controlled trial	958	Mean age 71 ± 11 years	CES-D (cutoff ≥ 16)	Composite of all-cause mortality and HF readmission over 18 months	
Wang et al. (43)	China	Multicenter prospective cohort study	4,130	Mean age 65.1 ± 13.5 years	PHQ-2 assessed before discharge and at 1 month after discharge	1-year cardiovascular death, all-cause death, HF rehospitalization, and all-cause rehospitalization	Adjusted HR
Xu et al. (44)	China	Prospective cohort study	146	Mean age 60 ± 12 years	HADS + HAMD (HADS-D ≥ 8 and HAMD ≥ 8)	1-year all-cause mortality and HF-related hospitalization	Adjusted HR

HF, heart failure; HR, hazard ratio; OR, odds ratio; BDI, beck depression inventory; DIS, diagnostic interview schedule; PHQ-2, patient health questionnaire-2; PHQ-9, patient health questionnaire-9; CES-D, center for epidemiologic studies depression scale; GDS-2, geriatric depression scale-2; GDS-15, geriatric depression scale-15; HADS, hospital anxiety and depression scale; HAM-D, Hamilton depression rating scale; ED, emergency department. Some publications originated from overlapping cohorts. These publications were included in the descriptive summary because they reported different outcomes, follow-up periods, or analyses. However, to avoid double-counting participants, only one independent effect estimate from each cohort was included in each pooled meta-analysis.

### Quality assessment

The methodological quality of the included studies was assessed using the Newcastle–Ottawa Scale (NOS). Among the 29 included studies, 23 were rated as high quality and 6 as moderate quality. The NOS scores ranged from 4 to 9, with a mean score of 7.30 and a median of 7, indicating that the overall methodological quality was generally acceptable.

Across domains, most studies performed well in the selection and outcome assessment domains. In particular, items such as S1, S2, S3, and O1 received relatively high scores, suggesting that the exposed cohorts were generally representative, the non-exposed groups were appropriately selected, exposure ascertainment was adequate, and outcomes were clearly defined and assessed.

However, lower scores were observed in the comparability and follow-up–related items, particularly C2, S4, and O3, indicating that some studies did not fully adjust for additional confounding factors or had limitations in baseline outcome assessment, follow-up duration, or completeness of follow-up.

In addition, the assessment of depression varied across studies, including self-reported scales, clinical diagnoses, and administrative coding, which may have introduced potential misclassification bias. Detailed quality assessment results are presented in Table 2.

**Table 2:** Methodological quality assessment of included studies using the Newcastle–Ottawa Scale (NOS).

Study	Selection (0–4)	Comparability (0–2)	Outcome/Exposure (0–3)	Total core (0–9)	Quality level
Jiang et al. (17)	3	1	3	7	High
Freedland et al. (18)	3	2	3	8	High
Lesman-Leegte et al. (19)	4	1	2	7	High
Lu et al. (20)	3	1	2	6	Moderate
Ma et al. (21)	4	2	3	9	High
Patel et al. (11)	3	0	2	5	Moderate
Piepenburg et al. (22)	3	1	3	7	High
Piepenburg et al. (23)	3	1	3	7	High
Reeves et al. (24)	3	2	2	7	High
Setoguchi et al. (25)	2	2	3	7	High
Song et al. (26)	3	1	3	7	High
Suzuki et al. (27)	4	2	3	9	High
Mao et al. (28)	4	2	3	9	High
Moraska et al. (29)	4	2	3	9	High
Suzuki et al. (30)	4	2	3	9	High
Albert et al. (31)	4	2	1	7	High
Alhurani et al. (32)	3	1	2	6	Moderate
Beach et al. (33)	3	1	3	7	High
Cha et al. (34)	4	2	2	8	High
Chavanon et al. (35)	4	2	3	9	High
Freedland et al. (36)	4	2	2	8	High
Oud et al. (37)	4	1	2	7	High
Redwine et al. (38)	4	1	2	7	High
Xu et al. (39)	3	0	1	4	Moderate
Zhang et al. (40)	4	1	3	8	High
Braunstein et al. (41)	3	1	2	6	Moderate
Jaarsma et al. (42)	4	2	0	6	Moderate
Wang et al. (43)	4	2	2	8	High
Xu et al. (44)	4	1	2	7	High

NOS, Newcastle–Ottawa Scale. Selection: 0–4 points; Comparability: 0–2 points; Outcome/Exposure: 0–3 points. Studies scoring 7–9 points were considered high quality, 4–6 points moderate quality, and 0–3 points low quality.

### Association between categorical depression and hospital readmission

Nine studies reporting multivariable-adjusted hazard ratios were included in the primary analysis. The pooled results indicated that depression was significantly associated with an increased risk of hospital readmission among older patients with heart failure (HR 1.76, 95% CI 1.30–2.40; *P* = 0.0003).

Considerable heterogeneity was observed across studies (*I*^2^ = 85%). Therefore, a random-effects model was applied (Figure 2).

**Figure 2:**
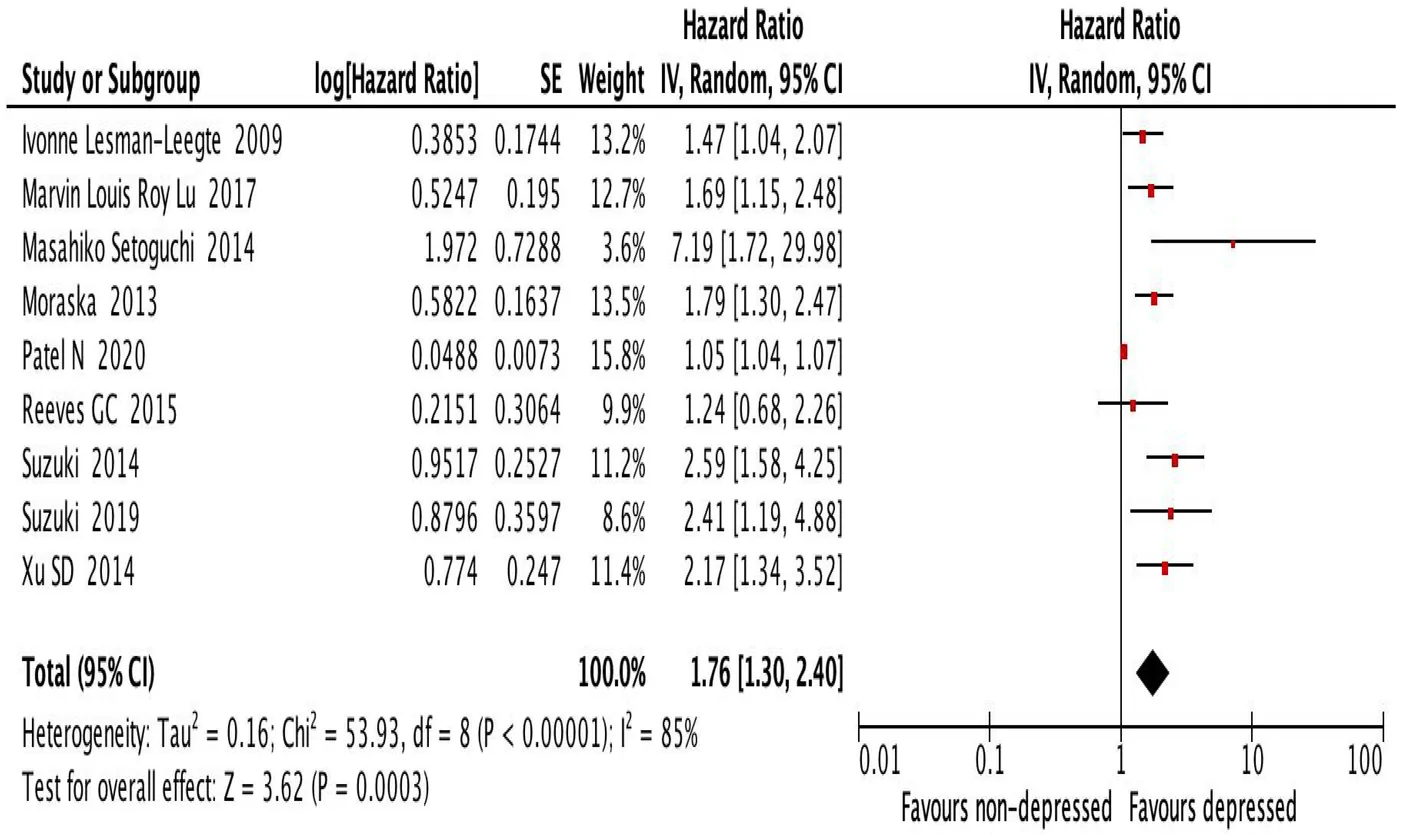
Forest plot of the association between categorical depression and hospital readmission based on multivariable-adjusted hazard ratios.

### Subgroup analyses by follow-up duration

Subgroup analyses were performed according to follow-up duration to explore potential sources of heterogeneity.

In studies with short-term follow-up (≤12 months), depression was not significantly associated with readmission risk (HR 1.49, 95% CI 0.92–2.40; *P* = 0.10), and substantial heterogeneity was observed (*I*^2^ = 82%).

In contrast, among studies with long-term follow-up (>12 months), depression was significantly associated with an increased risk of readmission (HR 1.87, 95% CI 1.43–2.44; *P* < 0.00001), with moderate heterogeneity (*I*^2^ = 45%).

However, the test for subgroup differences was not statistically significant (*P* = 0.41), suggesting that follow-up duration did not significantly modify the association between depression and readmission risk (Figure 3).

**Figure 3:**
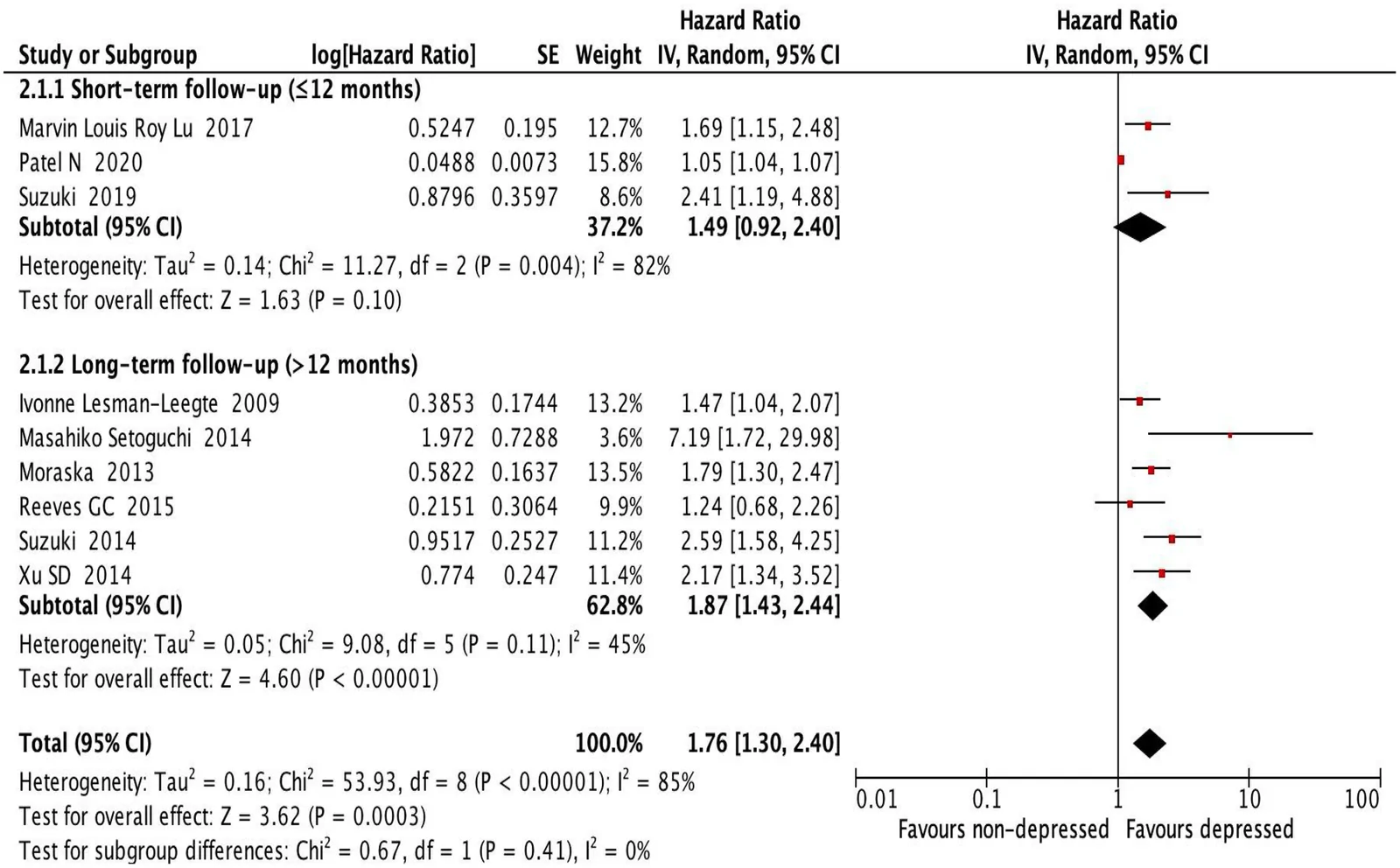
Subgroup analysis of the association between depression and adverse outcomes according to follow-up duration.

### Subgroup analysis by outcome type

Subgroup analyses were conducted according to outcome type.

For heart failure–related readmission, depression was significantly associated with an increased risk of readmission (HR 1.56, 95% CI 1.21–2.02; *P* = 0.0006), with no observed heterogeneity (*I*^2^ = 0%).

For composite outcomes including all-cause readmission or death, depression was also significantly associated with adverse outcomes (HR 1.87, 95% CI 1.25–2.81; *P* = 0.002), although substantial heterogeneity was present (*I*^2^ = 86%).

The test for subgroup differences was not statistically significant (*P* = 0.46), indicating that the association between depression and readmission risk did not significantly differ according to outcome type (Figure 4).

**Figure 4:**
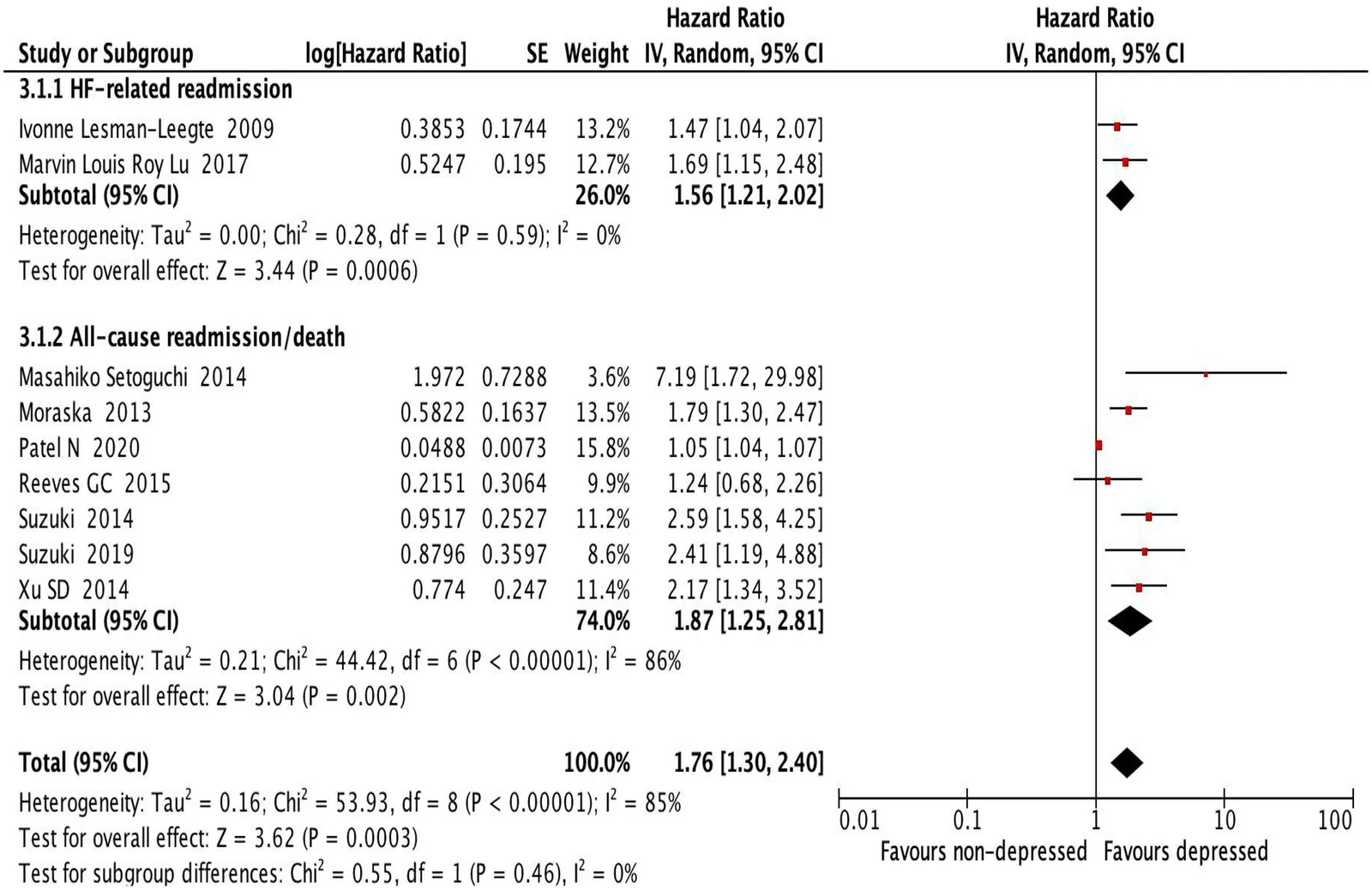
Subgroup analysis of the association between depression and hospital readmission according to outcome type.

### Subgroup analysis by depression assessment method

Subgroup analyses were conducted according to depression assessment method.

For studies using self-reported depression scales, depression was significantly associated with an increased risk of hospital readmission (HR 1.77, 95% CI 1.40–2.24; *P* < 0.00001), with low heterogeneity (*I*^2^ = 27%).

For studies using clinical diagnosis or administrative records, depression was also significantly associated with an increased risk of hospital readmission (HR 1.76, 95% CI 1.04–2.98; *P* = 0.03), although substantial heterogeneity was observed (*I*^2^ = 86%).

The test for subgroup differences was not statistically significant (*P* = 0.99), indicating that the association between depression and hospital readmission did not significantly differ according to the method used to assess depression (Figure 5).

**Figure 5:**
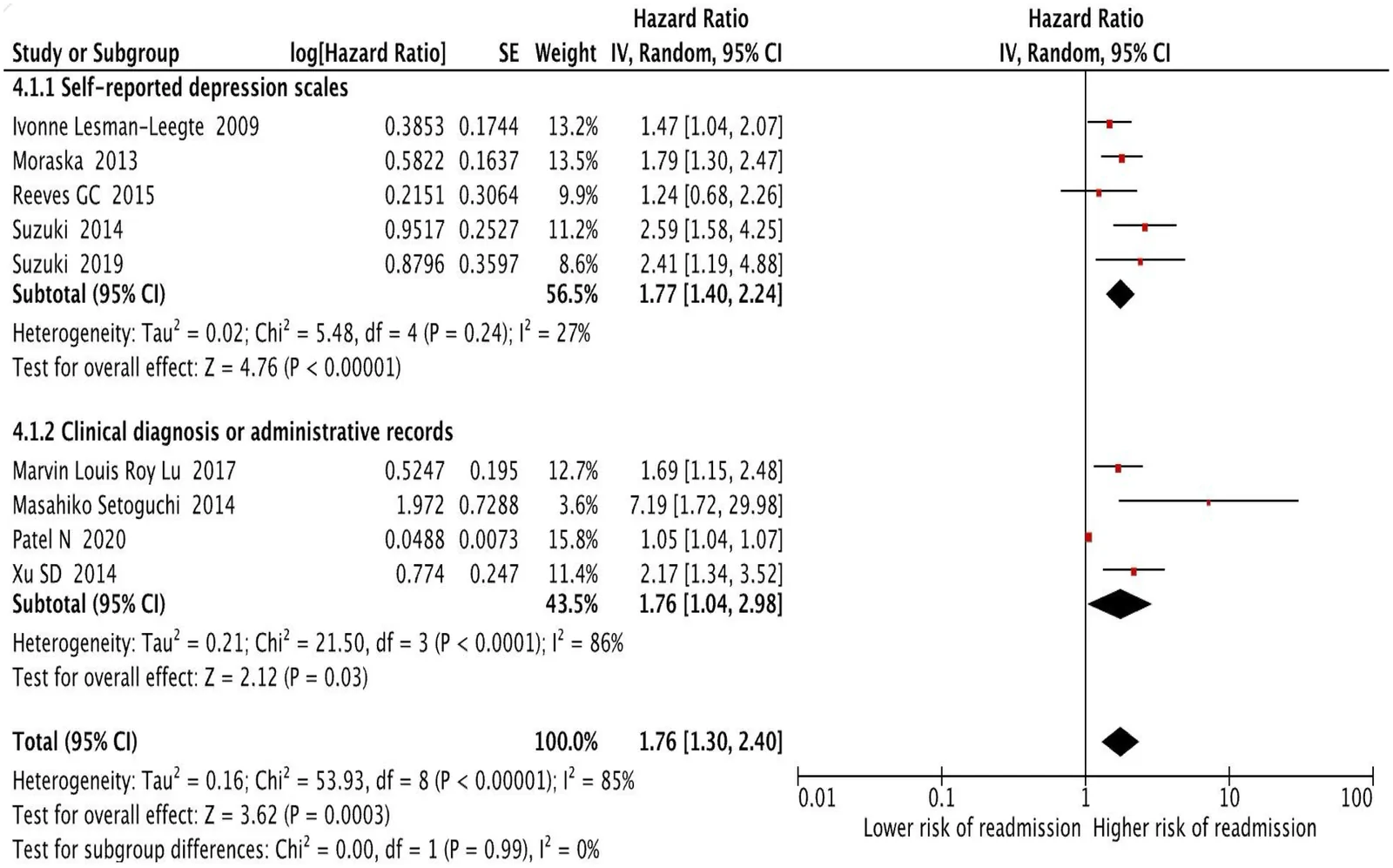
Subgroup analysis of the association between depression and hospital readmission according to depression assessment method.

### Association between continuous depression scores and readmission risk

Four studies reporting multivariable-adjusted hazard ratios based on continuous depression scores were included in the meta-analysis.

The pooled results showed that higher depression scores were significantly associated with an increased risk of hospital readmission among older patients with heart failure (HR 1.04, 95% CI 1.02–1.07; *P* < 0.0001).

Moderate heterogeneity was observed across studies (*I*^2^ = 55%). Therefore, a random-effects model was applied (Figure 6).

**Figure 6:**
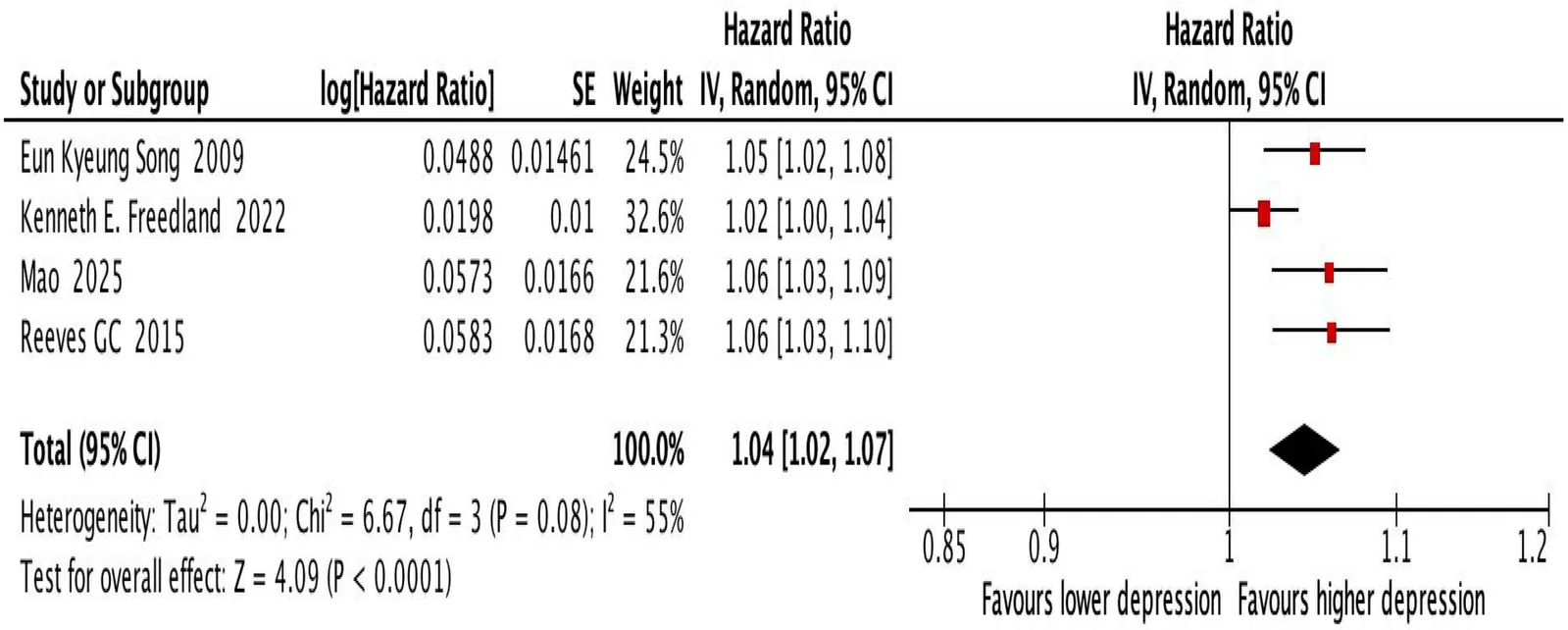
Forest plot of the association between continuous depression scores and hospital readmission based on multivariable-adjusted hazard ratios.

### Sensitivity analysis

To examine the robustness of the pooled results, sensitivity analyses were conducted using a leave-one-out approach by sequentially excluding each individual study.

In the analysis based on categorical depression, removal of any single study did not substantially alter the overall effect estimate. The pooled hazard ratios ranged from 1.66 to 1.85, and the association between depression and readmission risk remained statistically significant throughout the analyses.

A similar pattern was observed in the analysis of continuous depression scores. After excluding each study in turn, the pooled hazard ratios ranged from 1.04 to 1.06, with all estimates remaining statistically significant.

An additional sensitivity analysis restricted to studies with a mean age ≥65 years was also performed. The pooled hazard ratio was 1.66 (95% CI 1.17–2.35), and the association between depression and hospital readmission remained statistically significant.

These findings suggest that the observed association between depression and hospital readmission was stable and not driven by any single study. Similar results were observed after restricting the analysis to studies with a mean age of ≥65 years, suggesting that differences in age definitions across studies were unlikely to have materially influenced the primary findings.

### Publication bias

Publication bias was assessed visually using funnel plots (Figure 7). Visual inspection of the funnel plot showed no obvious asymmetry, suggesting that substantial publication bias was unlikely in the present analysis. However, as only nine studies were included in the pooled analysis, the ability to detect publication bias was limited and the results should therefore be interpreted with caution.

**Figure 7:**
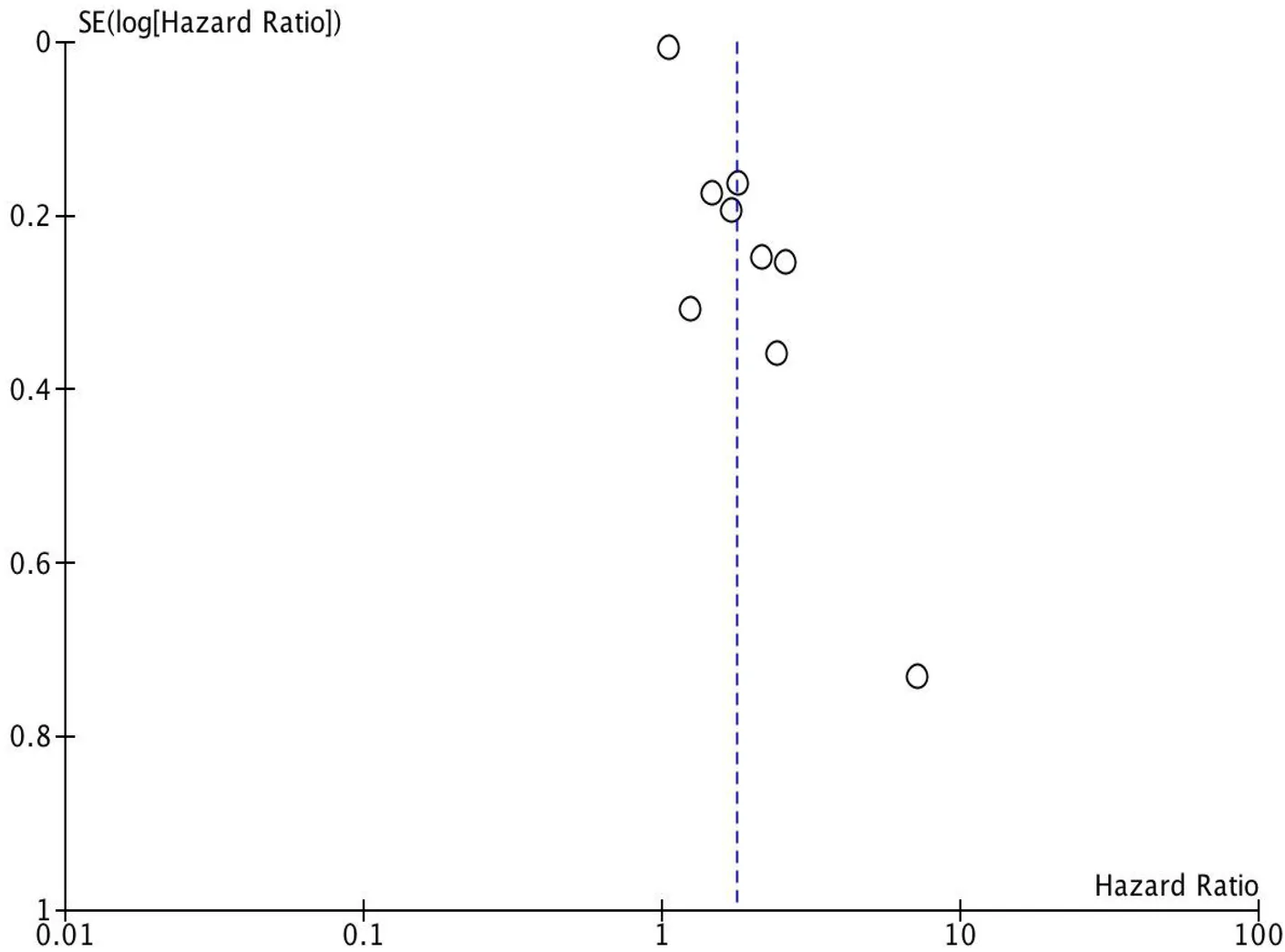
Funnel plot assessing publication bias for the association between categorical depression and hospital readmission.

## Discussion

### Summary of main findings

This systematic review and meta-analysis showed that depression was associated with a significantly increased risk of hospital readmission and other adverse clinical outcomes in patients with heart failure. In the pooled analysis of studies using categorical measures of depression, patients with depression had a 76% higher risk of adverse outcomes than those without depression. A similar pattern was observed in studies using continuous depression scores, where greater depressive symptom severity was also linked to poorer outcomes.

These findings suggest that the impact of depression on prognosis is not limited to its presence as a clinical condition, but may also extend across a gradient of symptom severity.

Importantly, the overall direction of the association remained broadly stable across subgroup analyses. This is consistent with earlier work showing that depression is associated with worse prognosis in heart failure populations (17). Subsequent prospective evidence has also supported depression as an independent predictor of adverse outcomes in patients with heart failure (36). Large-scale observational data have likewise shown that depression is associated with a higher risk of readmission after hospitalization (11).

Collectively, these findings reinforce the view that depression is not only clinically relevant, but may also represent a potentially modifiable factor in the course of heart failure.

Older adults with heart failure often face a particularly complex clinical situation, marked by multimorbidity, functional decline, and repeated hospitalization, all of which may intensify the impact of depression on post-discharge outcomes (40). Recent evidence has further shown that cardiovascular comorbidities are closely associated with psychological distress in older adults, highlighting the importance of integrating mental health assessment into routine cardiovascular care for this vulnerable population (10). In this context, depressive symptoms may be especially difficult to detect and manage, yet potentially more consequential. Recent evidence also suggests that depressive symptom burden remains clinically meaningful even in contemporary cohorts of older patients with heart failure (43).

This further highlights the importance of incorporating depression into routine risk assessment in this population.

### Comparison with previous studies

The present findings are broadly consistent with previous meta-analyses by Rutledge et al. (7) and Sokoreli et al. (8), both of which reported an association between depression and adverse outcomes in patients with heart failure. However, compared with these earlier reviews, the present study provides an updated synthesis by incorporating recently published cohort studies and evaluating depression using both categorical measures and continuous symptom scores. This approach provides a more comprehensive assessment of the relationship between depression and hospital readmission. Evidence from individual cohort studies further supports this association. For example, Lesman-Leegte et al. (19) reported that depressive symptoms were linked to an increased risk of both heart failure readmission and all-cause mortality. Similar associations have been observed in other studies, including the work of Suzuki et al. (27), who found a higher risk of heart failure-related readmission over 1 year, and Xu et al. (39), who reported poorer long-term outcomes, including mortality and heart failure-related events, among patients with depressive symptoms. Despite differences in study design and follow-up duration, these findings point to a broadly consistent pattern.

More recent evidence suggests that this relationship remains evident in contemporary clinical settings. Using a large administrative dataset, Patel et al. (11) found that depression was associated with an increased risk of both 30- and 90-day readmission. Findings from more recent cohorts also support this pattern. For instance, Wang et al. (43) highlighted the continued prognostic relevance of depressive symptoms in Chinese patients with heart failure, while Mao et al. (28) reported a similar association with cardiovascular-related readmission in older hospitalized patients. Overall, these studies suggest that the association between depression and adverse outcomes is not limited to specific populations or healthcare contexts.

At the same time, the strength of the association varied across studies, which may partly explain the heterogeneity observed in the present meta-analysis. One likely contributor is the method used to assess depression. Some studies relied on self-reported screening tools such as the PHQ-9, CES-D, or BDI (19, 27), whereas others identified depression based on clinical documentation or administrative data (11, 20). These differences may affect how depression is classified and could, in turn, influence the estimated effect sizes. Differences in adjustment strategies across studies may also explain part of the heterogeneity. Although adjusted hazard ratios were used in the meta-analysis, the variables included in the adjustment models differed between studies. Some studies adjusted only for basic demographic characteristics, whereas others also included disease severity, comorbidities, cardiac function, or treatment-related factors. These differences may partly explain the variation in the reported associations.

Differences in outcome definitions may have also played a role. While some studies focused specifically on heart failure-related readmission (20, 25), others examined broader outcomes such as all-cause readmission, mortality, or composite endpoints (24, 34). In addition, follow-up duration varied substantially across studies, ranging from 30 days to more than 1 year, which may further influence the observed associations. Even so, these methodological differences do not appear to alter the overall direction of the findings, which remains consistently aligned across studies.

### Potential mechanisms

Several mechanisms may explain the observed association between depression and the increased risk of re-hospitalization in elderly patients with heart failure. This relationship is likely to be multifactorial, arising from a combination of behavioral, clinical, and biological mechanisms rather than a single underlying cause (45). Depression may have a negative impact on patients' self-care behaviors and treatment compliance. After discharge, effective management of heart failure requires patients to follow the medication regimen, monitor symptoms, and maintain the recommended lifestyle changes (46). However, depressive symptoms such as lack of motivation, fatigue, and difficulty concentrating may reduce patients' ability to maintain these behaviors, thereby increasing the risk of worsening the condition and readmission (47).

Secondly, depression can affect symptom perception and the behavior of seeking treatment (48). Patients with depressive symptoms may have difficulty detecting the early signs of deterioration in their heart failure condition, or they may delay seeking medical attention (49). This delay can lead to more severe clinical symptoms when the patient is admitted again, especially in elderly individuals who already face challenges such as functional decline and complex care needs.

Thirdly, depression may indicate a broader clinical vulnerability. In elderly patients with heart failure, depressive symptoms often coexist with multiple diseases, functional impairments, and reduced social support (50). There may be interactions among these factors, leading to an increased likelihood of re-hospitalization. In such cases, depression not only may exist as an independent risk factor but also may play a role as an indicator of overall health status and the complexity of care.

Finally, biological mechanisms may also play a role. Depression is associated with dysregulation of the autonomic nervous system, activation of neuroendocrine pathways, and exacerbation of systemic inflammation (51). These processes can lead to worsening of the condition and a decline in physiological adaptability, thereby increasing the likelihood of adverse clinical outcomes (52).

However, given that most of the existing evidence comes from observational studies, these mechanisms should be understood as reasonable explanations rather than definite causal pathways.

### Clinical implications

These findings underscore the importance of considering depression in the clinical management of older patients with heart failure. Depression should not be merely regarded as a psychological complication, but should be considered as a factor with clinical relevance that may affect the prognosis of patients, especially in terms of the hospital readmission rate.

Recent evidence indicates that post-discharge care plays a crucial role in reducing the risk of readmission. A systematic review and meta-analysis reported that outpatient follow-up visits were associated with a 21% reduction in the risk of all-cause readmission within 30 days, and the effect was more significant in patients with heart failure (53). Structured follow-up and continuous monitoring after discharge are related to the improvement of treatment outcomes, especially in patients with heart failure. In clinical practice, such follow-up provides opportunities for optimizing medication, early detection of symptom deterioration, and strengthening self-management behaviors, which may be particularly beneficial for patients with depressive symptoms (54). From a nursing perspective, these findings emphasize the importance of incorporating psychological assessment into routine clinical care. Nurses, who are directly involved in the discharge planning, patient education, and post-discharge follow-up, are in a key position to identify depressive symptoms and provide early support. The improvement of self-management ability is related to the improvement of quality of life, and may help gradually alleviate depressive symptoms, which is very important for this population (55). In the clinical environment, continuous education and psychological support may be necessary, especially for elderly patients with heart failure. The participation of a multidisciplinary team, including professional nurses, may help strengthen self-management behaviors and improve long-term prognosis. Moreover, a single intervention measure is unlikely to fully meet the complex needs of these patients. Therefore, adopting a comprehensive approach that combines medical management, patient education, and psychosocial support may be more effective in reducing adverse consequences.

In addition, early identification of high-risk patients is essential for effective management (56). By using validated assessment tools for routine screening of depressive symptoms, clinicians can detect depression at an earlier stage and take timely intervention measures. This is a recommended practice in cardiovascular disease care, aiming to improve patient management and treatment outcomes (57). Incorporating depression into clinical risk assessment and stratification considerations may enable more effective identification of high-risk patients, as early identification has been proven to be crucial for formulating targeted management strategies (49). Given the multifactorial nature of readmission issues, a single intervention measure is unlikely to achieve the desired outcome. A comprehensive and multidisciplinary approach, including medical management, patient education, and psychosocial support, may be more effective in improving the long-term prognosis of elderly patients with heart failure (58).

### Strengths and limitations

This review has several strengths. It specifically addresses the relationship between depression and readmission in older patients with heart failure, an area that has received relatively limited attention in previous evidence syntheses. By including studies that assessed depression both categorically and as a continuum of symptom severity, the present analysis offers a broader view of how depression may relate to adverse clinical outcomes. The subgroup analyses also add some context to the findings, suggesting that the overall pattern of association remains relatively consistent across different follow-up periods and outcome definitions. In addition, the inclusion of recently published cohort studies provides an updated synthesis of the available evidence.

At the same time, a number of limitations should be acknowledged. Although the overall methodological quality of the included studies was generally acceptable, there was noticeable variation in the way confounding factors were handled. Some studies adjusted for only a limited number of covariates, whereas others used more comprehensive adjustment models. This makes direct comparison more difficult and leaves open the possibility of residual confounding.

There was also inconsistency in how baseline status and follow-up were reported across studies. In some cases, it was not entirely clear whether the outcome of interest was absent at baseline, and the duration or completeness of follow-up was not always comparable. These differences may have contributed to the heterogeneity observed in the pooled analysis. The definition of older adults was not consistent across the included studies because each study applied its own eligibility criteria. We therefore performed a sensitivity analysis restricted to studies with a mean age of ≥65 years. The association remained statistically significant, suggesting that differences in age criteria did not materially affect the overall findings.

Another limitation relates to the measurement of depression. The included studies used a range of approaches, including self-reported questionnaires, clinical diagnoses, and administrative coding. Although each of these methods has its own value, the lack of consistency across studies makes the findings more difficult to interpret in a fully uniform way.

Finally, because most of the included studies were observational in design, the results should be interpreted as indicating association rather than causation. Taken together, these limitations do not negate the overall findings, but they do suggest that the results should be interpreted with appropriate caution, particularly when drawing clinical or causal inferences.

## Conclusion

Overall, the findings of this review suggest that depression is associated with a higher risk of readmission and other adverse outcomes in older patients with heart failure. This relationship was observed not only when depression was defined as a categorical condition, but also when depressive symptoms were considered in terms of severity. Although some heterogeneity across studies should be acknowledged, the overall pattern of evidence indicates that depression is a clinically relevant factor in the prognosis of this population.

From a clinical perspective, these findings underscore the need to pay greater attention to depression in the routine management of older adults with heart failure, particularly during the period following hospital discharge. Better recognition of psychological symptoms may help improve risk identification, support more individualized care, and contribute to the development of future strategies aimed at reducing preventable readmissions.
