# Supplementary material for: Depression and risk of hospital readmission in older patients with heart failure: a systematic review and meta-analysis
Source: Front Cardiovasc Med. 2026 Sep 18;13:1917904. doi: 10.3389/fcvm.2026.1917904 (PMC13631436; doi:10.3389/fcvm.2026.1917904)
Supplement: Supplementary file 1 [file Table1.docx]

Supplementary Table S1. Detailed search strategies for each database

| **Database** | ****Coverage**** | **Search strategy** |
| --- | --- | --- |
| Pubmed | Coverage：Database inception to 31 December 2025 | (“heart failure”[Title/Abstract]  OR “cardiac failure” [Title/Abstract] OR “congestive heart failure”[Title/Abstract]) AND (depression[Title/Abstract] OR “depressive symptoms”[Title/Abstract] OR depressive[Title/Abstract]) AND (readmission[Title/Abstract] OR rehospitalization[Title/Abstract] OR “hospital readmission”[Title/Abstract]) |
| Embase | Coverage：Database inception to 31 December 2025 | (‘heart failure’:ti,ab OR ‘cardiac failure’:ti,ab OR ‘congestive heart failure’:ti,ab) AND (‘depression’:ti,ab OR ‘depressive symptoms’:ti,ab OR ‘depressive’:ti,ab) AND (‘readmission’:ti,ab OR ‘rehospitalization’:ti,ab OR ‘hospital readmission’:ti,ab) |
| Web of Science | Coverage：Database inception to 31 December 2025 | TS=(“heart failure” OR “cardiac failure” OR “congestive heart failure”) AND TS=(depression OR “depressive symptoms” OR depressive) AND TS=(readmission OR rehospitalization OR “hospital readmission”) |
| CNKI | Coverage：Database inception to 31 December 2025 | （心力衰竭 OR 心功能不全）AND（抑郁 OR 抑郁症状）AND（再入院 OR 再住院 OR 再住院率） |
| WanFang Data | Coverage：Database inception to 31 December 2025 | （心力衰竭 OR 心功能不全）AND（抑郁 OR 抑郁症状）AND（再入院 OR 再住院 OR 再住院率） |

**Note:** Literature searches were conducted from database inception to **31 December 2025**. Reference lists of eligible studies and relevant reviews were manually screened to identify additional eligible studies. Only peer-reviewed studies were considered eligible for inclusion. **Preprint servers were not searched.**
